# Automated Detection, Localization, and Severity Assessment of Proximal Dental Caries from Bitewing Radiographs Using Deep Learning

**DOI:** 10.3390/diagnostics15070899

**Published:** 2025-04-01

**Authors:** Mashail Alsolamy, Farrukh Nadeem, Amr Ahmed Azhari, Walaa Magdy Ahmed

**Affiliations:** 1 Department of Information Systems, Faculty of Computing and Information Technology, King Abdulaziz University, Jeddah 22233, Saudi Arabia; 2 Department of Restorative Dentistry, Faculty of Dentistry, King Abdulaziz University, Jeddah 22233, Saudi Arabia

**Keywords:** proximal caries, YOLO network, diagnosis, artificial intelligence, bitewing radiographs, instance segmentation

## Abstract

**Background/Objectives**: Dental caries is a widespread chronic infection, affecting a large segment of the population. Proximal caries, in particular, present a distinct obstacle for early identification owing to their position, which hinders clinical inspection. Radiographic assessments, particularly bitewing images (BRs), are frequently utilized to detect these carious lesions. Nonetheless, misinterpretations may obstruct precise diagnosis. This paper presents a deep-learning-based system to improve the evaluation process by detecting proximal dental caries from BRs and classifying their severity in accordance with ICCMS^TM^ guidelines. **Methods**: The system comprises three fundamental tasks: caries detection, tooth numbering, and describing caries location by identifying the tooth it belongs to and the surface, each built independently to enable reuse across many applications. We analyzed 1354 BRs annotated by a consultant of restorative dentistry to delineate the pertinent categories, concentrating on the detection and localization of caries tasks. A pre-trained YOLOv11-based instance segmentation model was employed, allocating 80% of the dataset for training, 10% for validation, and the remaining portion for evaluating the model on unseen data. **Results**: The system attained a precision of 0.844, recall of 0.864, F1-score of 0.851, and mAP of 0.888 for segmenting caries and classifying their severity, using an intersection over union (IoU) of 50% and a confidence threshold of 0.25. Concentrating on teeth that are entirely or three-quarters presented in BRs, the system attained 100% for identifying the affected teeth and surfaces. It achieved high sensitivity and accuracy in comparison to dentist evaluations. **Conclusions**: The results are encouraging, suggesting that the proposed system may effectively assist dentists in evaluating bitewing images, assessing lesion severity, and recommending suitable treatments.

## 1. Introduction

Dental caries are among the most widespread and prevalent dental diseases globally. The progressive nature of caries highlights the essential requirement for early detection since prompt intervention can greatly reduce tooth damage and the ensuing need for extensive therapies [1]. Timely detection of carious lesions allows healthcare providers to employ preventive measures, including fluoride treatments and minimally invasive restorative techniques, thus safeguarding tooth structure and function [2]. Moreover, managing caries in their early phases not only improves individual dental health results but also alleviates the overall strain on healthcare systems by reducing the demand for time-consuming and expensive complicated treatments like root canals or extractions. This highlights the necessity of developing clinical decision-support tools to aid professionals in evaluating these lesions.

Visual inspection and radiographic analysis are typically employed in the diagnosis of carious lesions. Imaging techniques, particularly bitewing radiographs (BRs), are essential for the identification of interproximal caries, as the location of these lesions makes them challenging to detect clinically. BRs provide a focused perspective, facilitating a more precise evaluation of interproximal decay. They typically cover the area from the distal surface of the canine teeth to the distal surface of the last molar. Dental lesions are depicted as a radiolucent region in bitewing films, which is indicative of their diminished X-ray absorption [3]. However, the assessment of lesion depth using radiographic examination may demonstrate low sensitivity, particularly for early-stage lesions [4]. In addition, the clarity of BRs can be influenced by various factors, including noise and artifacts, overlapping structures, and cervical burnout [5], which may obfuscate the diagnosis. Consequently, the reliability of evaluations can be substantially improved by implementing supplementary methods incorporating computational analysis.

In recent years, artificial intelligence algorithms, particularly deep learning (DL) methods, have acquired traction in the field of dental medicine, yielding impressive outcomes [6,7,8,9]. The application of convolutional neural networks (CNNs) has been effective in the identification of a variety of lesions when analyzing radiographs as a supplementary diagnostic instrument such as periodontal bone loss [10], apical lesions [11], and maxillary sinusitis [12]. CNNs are a distinct category of DL algorithms specifically engineered to proficiently evaluate visual data, especially images, by autonomously extracting hierarchical characteristics across numerous processing layers. These networks employ convolutional layers to identify patterns and characteristics, rendering them exceptionally successful for computer vision tasks [13].

The detection of caries has been effectively handled by diverse strategies, including classification, object detection, and segmentation techniques. Classification yields a binary presence/absence determination of caries, object detection facilitates spatial localization by generating a Bbox around the area of interest, and segmentation provides intricate delineation of carious lesions by highlighting the targeted region, each contributing distinctly to the thorough evaluation of dental caries.

Classification: Mao et al. [14] developed a model to classify the restoration and caries. Several image-processing techniques were employed to isolate 700 teeth retrieved from 278 BRs, comprising 350 targeted and 350 non-targeted specimens. Each tooth was subsequently split in half and utilized as an input for the classification model. Four DL networks were assessed, ResNet50, GoogleNet, Vgg19, and AlexNet, where the latter achieved the best accuracy results of 95.56%. Moran et al. [15] also used image processing methods to prepare the data for the classification model by extracting the teeth from 112 BRs. Each tooth was subsequently labeled by one of three classes: normal (305 teeth), incipient caries (113 teeth), and advanced caries (62 teeth). The study employed DL-based classifiers, Inception and ResNet, with Inception achieving the highest accuracy of 73.3%.

Object detection: Object-detection-based models are typically classified into two main categories: one- and two-stage models. One-stage models execute object detection in a singular pass, such as YOLO and SSD. The model concurrently predicts Bboxes and class probabilities from the entire image, resulting in increased processing speed. In contrast, two-stage models initially produce region proposals, followed by the refinement of these proposals to classify objects and predict their Bboxes, resulting in a slower speed but higher accuracy, such as R-CNN, Fast R-CNN, and faster R-CNN. Several studies used YOLO of different versions to detect caries from BRs. Bayraktar and Ayan [16] employed YOLOv3 to detect proximal caries without assessing their severity. A total of 1000 BRs were utilized to train and assess the model, which achieved a precision of 0.866 and a recall of 0.723. The YOLOv3 was also used in the study [17] to detect caries and identify their severity according to ICCMS^TM^. The BRs were annotated by drawing a Bbox around the tooth and categorizing it into one of the following classes: non-carious, initial1, initial2, initial3, moderate4, extensive5, and extensive6. This annotation method can lead to confusion when two different cases of caries with varying severity are present on the same tooth. The same method of annotation was followed in a later study [18] that utilized a pre-trained YOLOv7. The authors also assessed the performance of YOLOv3. The findings indicated that YOLOv7 had enhanced performance relative to YOLOv3. In the study [19], a Faster R-CNN was used to detect proximal caries, attaining precision, recall, and F1-score values of 0.86, 0.89, and 0.87, respectively, without considering caries severity. Ayhan et al. introduced an innovative model that concurrently detects proximal caries and enumerates teeth utilizing YOLOv7 [20]. The F1-score, precision, and recall of matching the caries area with tooth number were 0.842, 0.851, and 0.834, respectively. However, the severity of carious lesions was not considered.

Segmentation: Several studies in the literature used U-Net architecture to segment caries by highlighting targeted objects. The U-Net was designed specifically for semantic segmentation. Semantic segmentation aims to categorize each pixel in an image into a distinct class [21]. Lee et al. designed a U-Net-based model for the segmentation of dental structures, including enamel, dentin, pulp, restoration, gutta-percha, and caries [22]. A total of 354 BRs were utilized. The model achieved precision, recall, and F1-score values of 0.633, 0.65, and 0.641, respectively. Despite the inclusion of various caries forms, the severity of the caries was not assessed. Cantu et al. [23] used U-Net to detect caries of different severity. The model achieved a precision of 0.7 and a recall of 0.75. However, the severity of caries was not distinguished in the output where all predicted caries were represented by the same color. In contrast, a study published by Ahmed et al. added a further procedure to classify the severity of caries into five categories by employing three different encoders: Vgg19, ResNet50, and ResNext101 [24]. Their proposed model attained a mean F1-score of 0.612 and a mean IoU of 0.566, which ResNext101 achieved.

YOLO, an acronym for “You Only Look Once”, is one of the most well-known rapid object recognition algorithms. Its purpose is to efficiently detect and localize objects in images or video by generating bounding boxes (Bboxes) surrounding them [25]. The latest versions of YOLO have enhanced their functionalities to encompass a wider array of computer vision tasks beyond conventional object detection, such as instance segmentation, pose/keypoints, and classification. The YOLO-based instance segmentation enhances the conventional YOLO object detection method by not only recognizing objects in an image but also accurately outlining their shapes. The design of YOLO was modified to predict both Bboxes and pixel-level masks for each identified object. This entailed incorporating further output layers that precisely manage mask predictions. For each identified object, the model produces a binary mask that delineates the pixels associated with that object. This is generally accomplished by a CNN that learns to generate these masks during training. Throughout training, the model acquires the ability to optimize both the Bbox coordinates and the associated masks. During inference, when an image is processed by the model, it generates Bboxes accompanied by their respective confidence scores and masks. The anticipated masks are subsequently applied to the Bboxes to provide accurate segmentations of each object. Ultimately, post-processing techniques like non-maximum suppression (NMS) are employed to enhance the predictions, assuring proper management of overlapping boxes and precise representation of each object. By including these procedures, YOLO can proficiently execute instance segmentation, delivering comprehensive information regarding the existence and form of objects within an image.

**Figure 1:**
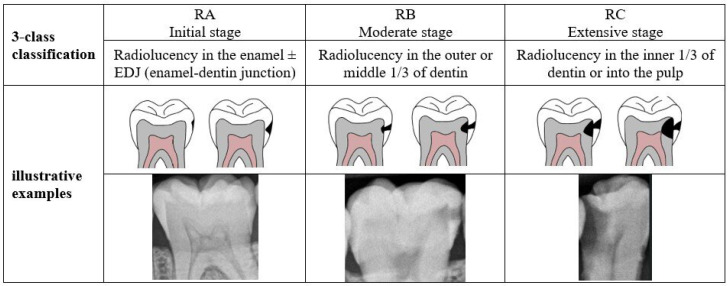
Classes description of caries evaluated in the study according to ICCMS^TM^.

To the best of our knowledge, no research exists that identifies caries of varying severity while offering adequate diagnosis, including the number of affected teeth and the impacted surfaces. The previous studies are restricted to detecting caries and determining their severity in a few cases which is insufficient for dentists to complete the dental chart. Therefore, this study aims to develop a novel proximal caries diagnostic system based on deep learning (DL) that provides a comprehensive and accurate diagnosis of proximal caries, including the severity of the caries, the affected tooth, and the involved surface. This study aims to develop a novel proximal caries diagnostic system based on DL that delivers accurate diagnoses of proximal caries, including the severity of caries, the tooth affected, and the surface involved. To achieve this, the proposed system integrates three main tasks: detecting caries, numbering teeth, and identifying affected teeth and surfaces. The purpose of this system is to assist dentists, rather than replace them, in providing accurate and efficient caries diagnosis. The severity of caries follows the International Caries Classification and Management System (ICCMS^TM^) [3] which categorizes the stages of carious lesions into three main categories: initial, moderate, and extensive. The details of these stages are presented in Figure 1. A recent iteration of YOLO-based instance segmentation, in short YOLOv11-seg, is used to identify proximal caries and their severity. The effectiveness of the system is assessed by comparing its performance to that of dentists.

## 2. Materials and Methods

This work was authorized by the Research Ethics Committee (approval no. 367-12-21, 31 January 2022), Faculty of Dentistry, King Abdulaziz University, KSA.

### 2.1. Data Collection

A total of 1354 digital BRs were retrieved from the archive of the University Dental Hospital of King Abdulaziz University from 2020 to 2022 in the RadioVisioGraphy (RVG) format and size 3300 × 2550. RVG is a digital X-ray technology that is widely regarded as one of the most cutting-edge technological advancements in the field of dentistry. This approach utilizes electronic sensors to capture digital films, replacing the outdated film shooting technique. The BRs were picked without considering the patient’s age, gender, or clinical information. The exclusion criteria were BRs depicting primary teeth, with no caries, and blurred images; otherwise, they were included. The data were taken by the Care Stream CS2100 intra-oral X-ray generator. All images were saved in JPG format and anonymized to safeguard patients’ privacy. The BRs were subsequently increased by implementing augmentation techniques: vertical and horizontal flips. As a preprocessing step, the images were resized to 1024 × 768 to obtain a suitable scale for training while preserving the resolution of caries.

### 2.2. Image Annotation

Before annotation, the images were enhanced by applying the contrast limited adaptive histogram equalization (CLAHE) technique to improve the visibility of carious lesions for annotators. The clipLimit parameter was set to 1.4 with a tileGridSize of (8,8). The caries annotation procedure was conducted consensually by two experts, each with at least 7 years of experience. A polygonal shape was delineated around each proximal carious lesion, labeled into one of three classes illustrated in Figure 1 using the CVAT annotation tool. To ascertain the characteristics of each class, the specialists adhered to predetermined guidelines to guarantee consistency and precision in classification. In instances where the two annotators disagreed, a third dentist was consulted to render the final decision, thereby increasing the reliability of the annotations. The accuracy of the segmentation was enhanced by this collaborative approach, which also ensured the dataset’s credibility for future analysis. The annotation was exported in COCO format to construct the reference datasets for the segmentation and object detection tasks that were compatible with the YOLO network. This format structures image annotations within a JSON file containing both the bounding box and segmentation coordinates for each labeled caries. This enables us to retrieve annotations in desired formats. The first format was created as a discrete text file for each image, which includes the class ID followed by the x and y coordinates of the polygon for each caries; the class ID is 0 for RA, 1 for RB, and 2 for RC. These text files constructed the reference dataset for the YOLO-based segmentation task. The second format was created similarly to the first, with the polygon’s coordinates substituted by the x-center, y-center, width, and height of the bounding box (Bbox) to form the YOLO-based object detection task reference dataset.

### 2.3. Proximal Caries Diagnostic System (PCDS) Design

**Figure 2:**
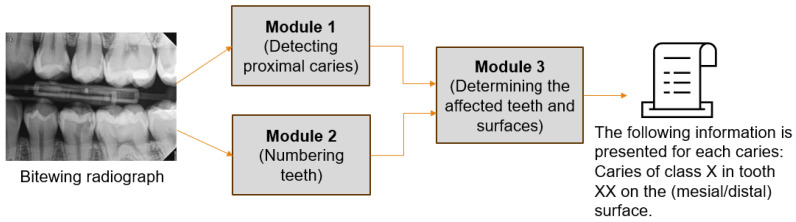
The framework of the proximal caries diagnostic system (PCDS).

This system consists of three distinct modules, each performing a specific task, as shown in Figure 2. Because of this, the modules can be reused in various applications. The modules are detecting proximal caries, numbering teeth, and determining the affected teeth and surfaces, Module1 is invoked initially by the system, followed by module2 in the event that caries are detected. Module3 then incorporates the results of the two modules to generate the final diagnosis. This study addresses module1 and module3 while module2 was implemented in our previous study [26] published in October 2024.

#### 2.3.1. Caries Detection Module

This module integrates with other modules to build the system of proximal caries diagnosis. Consequently, the selected approach must consider the techniques employed in other modules to ensure their seamless integration into a cohesive system.

**Figure 3:**
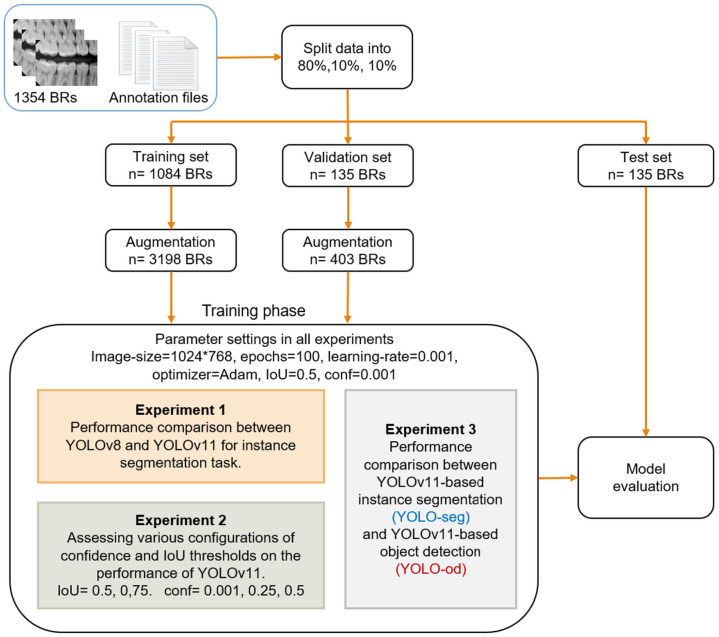
Schema of the methodology conducted in caries detection module.

The methodology employed in this module is depicted in Figure 3. The dataset was split into training, validation, and test sets in the ratios of 80%, 10%, and 10%, respectively. This results in 1084 BRs allocated for training the model and 135 BRs to evaluate the model during training and after each epoch to help in tune the model’s hyperparameters and detect overfitting. The training and validation sets were expanded to provide 3198 BRs and 403 BRs, respectively. A total of 135 BRs of the test set utterly unseen by the model were used to give a final, unbiased evaluation of the model’s performance.

In the training phase, three experiments with different objectives were conducted utilizing a state-of-the-art version of the YOLO network-based instance segmentation technique (YOLOv11-seg) to perform caries detection and segmentation. Instance segmentation integrates the characteristics of object identification and semantic segmentation techniques by localizing each object with a bounding box and supplying a pixel-level mask within each box [27]. It is advantageous for addressing overlapping objects, ensuring each object is differentiated from the others.

In all experiments, the YOLO model was configured with these parameters: training the model for 100 epochs, batch size of 8, image size of 1024 × 768, and using Adam optimizer at a 0.001 learning rate.

**Experiment 1: Comparison of YOLOv8 and YOLOv11**

YOLOv11 is a recent generation of YOLO designed to surpass the performance of its predecessors. This experiment aims to examine the capabilities of YOLOv11 by comparing its performance with its predecessor, YOLOv8, in the instance segmentation challenge under the default settings of IoU and confidence thresholds.

**Experiment 2: Performance evaluation of YOLOv11 based on IoU and confidence threshold variations**

**Figure 4:**
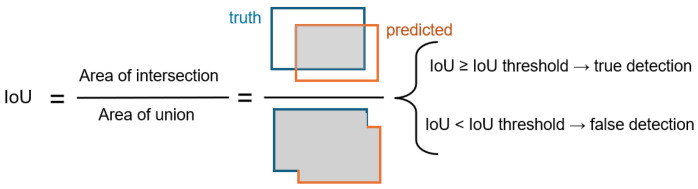
Intersection over union equation.

Intersection over union (IoU) and confidence (conf) thresholds are crucial parameters in YOLO that influence the final predictions, and their impact was assessed in this experiment. The IoU signifies the detection area that must closely approximate the expert-derived ground truth, as shown in Figure 4. In contrast, the confidence threshold establishes the minimum confidence score necessary for a predicted mask to be deemed valid. If the confidence threshold is established at 0.5, only masks with confidence scores over this level will be regarded as valid detections. We performed the experiment using two distinct IoU thresholds: 50% and 75%. We assessed the model’s performance at each IoU level using three distinct confidence thresholds: 0.001, 0.25, and 0.5. The default configuration for the YOLO model was a confidence threshold of 0.001 and an IoU of 50%. By systematically varying these parameters, an evaluation of their influence on detection accuracy and overall model effectiveness was considered. The findings of this experiment offer significant insights into how threshold modifications can enhance instance segmentation efficacy in real-world applications.

**Experiment 3: Comparison of YOLOv11 for instance segmentation and object detection**

This study posited that instance segmentation is superior to object detection in the context of caries detection, as segmentation offers pixel-level classification, facilitating accurate localization and identification of carious lesions, essential for various stages of caries. To substantiate this assertion, a performance comparison between the two approaches in this experiment was executed.

The Wilcoxon signed ranks test was employed to compare models’ performance in each experiment based on the mAP50 metric, with a *p*-value of less than 0.05 being statistically significant.

#### 2.3.2. Determining the Affected Teeth and Surfaces Module

**Figure 5:**
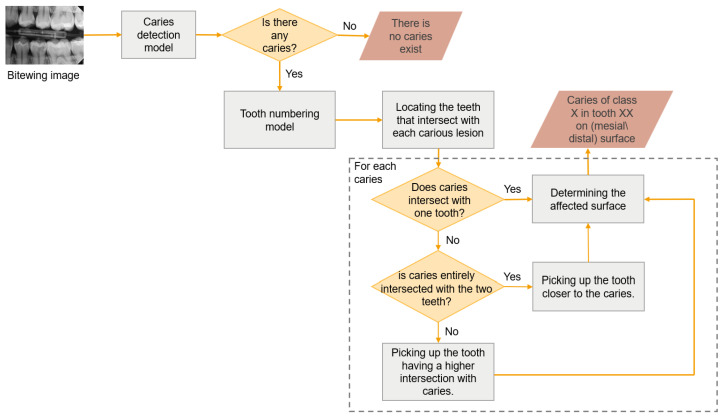
The workflow of the proximal caries diagnostic system.

This module integrated the outcomes of PCDM and the tooth numbering model to identify the affected teeth and surfaces, as shown in Figure 2. The outcomes of the tooth numbering model are the Bbox coordinates per tooth along with its number. The benefit of the instance segmentation method is the incorporation of segmentation masks in conjunction with Bboxes. Therefore, this module’s work is predicated on the coordinates of Bboxes for teeth and caries. As both models were derived from the YOLO network, their findings were seamlessly integrated without necessitating any alterations to their format. The procedures followed in this module were illustrated in Figure 5. For each carious lesion, the intersecting teeth are identified, resulting in one of three scenarios:

- The caries was overlapped with one tooth, classified as the affected tooth.
- The caries was overlapped with two teeth; however, one tooth exhibited a greater degree of overlap than the other and was therefore classified as the affected tooth.
- The caries was overlapped completely with two teeth; in this case, the tooth where the caries is closest to its Bbox edge was classified as the affected tooth.

After that, the surface was determined by comparing the x-midpoint of the caries’s Bbox with the x-midpoint of the affected tooth’s Bbox, considering the side of the BR, whether left or right.

All tasks were performed on a PC equipped with an NVIDIA GeForce RTX 4070 GPU and 32 GB of RAM. All the code was written in Python and executed on the JupyterLab platform using Python 3.8. The Ultralytics-8.3.23 was utilized to access and execute the YOLO network.

### 2.4. Performance Assessment

Selection of appropriate metrics that accurately reflect performance is crucial for any experiment’s performance evaluation. Metrics such as precision, recall, F1-score, and mAP are frequently employed in detection and segmentation tasks and were assessed in this study. The computation of these metrics relies on the quantities of true positive (TP), false positive (FP), and false negative (FN) instances. This entails computing the IoU between predicted and actual instances and comparing it with the IoU threshold, as shown in Figure 4. A TP denotes the instances detected by the model that correspond with the reference dataset while FP means an instance was detected but does not correspond with the reference dataset. This entails computing the IoU between predicted and actual. Conversely, a FN signifies the instances not recognized by the model and in the reference dataset.

Precision denotes the ratio of accurately identified instances to the total detected instances while recall reflects the model’s ability to recognize all relevant instances in the reference dataset. When both precision and recall are significant, the F1-score offers a single metric that equilibrates the two.

The mAP is an essential evaluation statistic employed in YOLO and other object detection models to evaluate their performance across various classes. It computes the average precision (AP) for each class across various IoU thresholds, offering a thorough assessment of the model’s proficiency in reliably detecting and localizing objects, as in Equation (1), where C is the number of classes. It provides a singular metric that encapsulates both precision and recall of the model, facilitating performance comparisons among various models and settings. An elevated mAP signifies enhanced overall detection precision and resilience in recognizing objects under varied situations.

(1) $$\mathrm{mAP}=\frac{1}{C}\sum_{i=1}^{C}{\mathrm{AP}}_{i}$$

In this study, the mAP was computed under the $IoU_{50}$ threshold and denoted mAP50, which means the area under the curve between precision and recall at IoU is equal to 0.5. The effectiveness of the system was evaluated by comparing its results of 10 BRs with those of seven general dentists with varying levels of experience.

## 3. Results

Table 1 displays the quantitative statistics for each dataset and category.

### 3.1. Experiment 1: Performance Comparison of YOLOv8 and YOLOv11 of Instance Segmentation Task Under Similar Conditions

**Figure 6:**
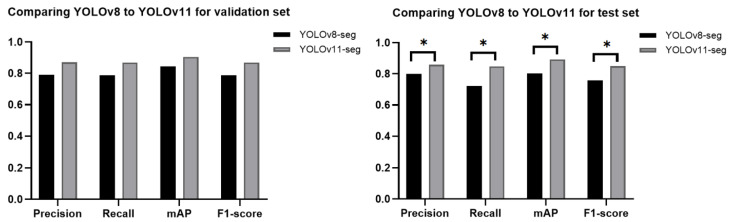
The outcomes of the validation and test datasets for YOLOv8-seg and YOLOv11-seg. The asterisk (*) signifies a statistically significant enhancement attained by YOLOv11-seg.

The YOLOv11-seg showed significant improvement in all evaluation metrics compared to the YOLOv8-seg as presented in Figure 6; the *p*-value of 0.028 proved this significant. The results indicated that the capabilities of YOLOv11-seg surpass those of its predecessors, prompting our decision to utilize YOLOv11-seg.

### 3.2. Experiment 2: YOLOv11 with Different IoU and Confidence Thresholds

**Figure 7:**
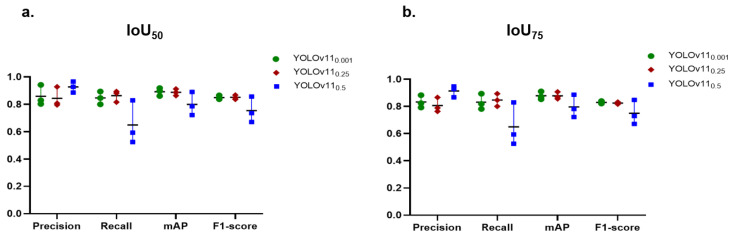
A performance of YOLOv11-seg on the test set under $IoU_{0.5}$ (**a**) and $IoU_{0.75}$ (**b**) with varying confidence threshold values. The horizontal line denotes the mean of the three classes.

**Figure 8:**
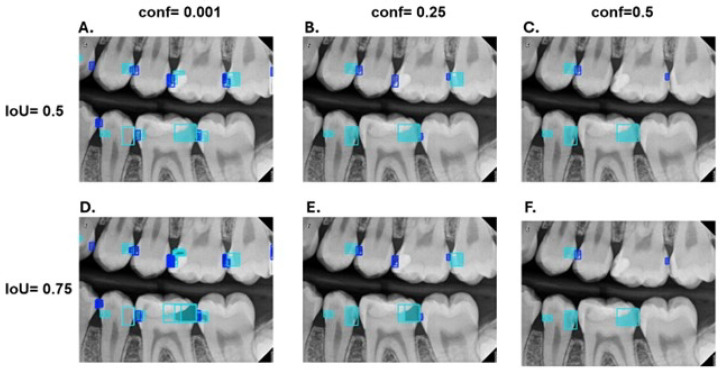
The results of PCDM under different values of IoU and confidence thresholds; where class RA is represented in (blue), RB in (turquoise), and RC in (lightgray).

In this experiment, the performance of YOLOv11-seg was assessed under $IoU_{50}$ and $IoU_{75}$ and confidence thresholds at 0.001, 0.25, and 0.5. The scatter plot in Figure 7 shows the performance comparison of the test set while Figure 8 represents the predicted caries across these values.

#### 3.2.1. Changing IoU Thresholds, $IoU_{50}$ vs. $IoU_{75}$

Generally, precision scores are higher at $IoU_{50}$, indicating that more predictions are accepted as TP at this permissive threshold. This results in elevated recall rates as well. At $IoU_{75}$, both precision and recall exhibit a decline, signifying that the more stringent threshold complicates the model’s ability to satisfy the requirements for true positives, particularly impacting recall in specific classes. Statistically, there is a significant difference in the mAP50 of YOLOv11-seg at $IoU_{50}$ compared to $IoU_{75}$ in the three values of confidence ($YOLOv11_{0.001}$ and $YOLOv11_{0.25}$, *p* = 0.027; $YOLOv11_{0.5}$, *p* = 0.042).

#### 3.2.2. Confidence Threshold Variations (0.001; 0.25; 0.5)

In general, the number of predictions increases as the confidence threshold is reduced (0.001, 0.25). This can increase recall, but it may also reduce precision due to the increased number of FPs. Conversely, an increasing confidence threshold to 0.5 typically results in a higher precision but a lower recall. The model is at risk of overlooking genuine positive cases as it becomes more selective in its classification of positive predictions.

As a result, the combination of an IoU of 0.5 and a confidence threshold of 0.25 appears to provide the best equitable performance for the segmentation of carious lesions, as demonstrated in Figure 8, where low confidence generates multiple bounding boxes for the same caries, while high confidence leads to the omission of certain true instances. The performance of PCDM under these settings is presented in Table 2.

### 3.3. Experiment 3: Performance Comparison Between Object Detection and Instance Segmentation Tasks Based on YOLOv11

**Figure 9:**
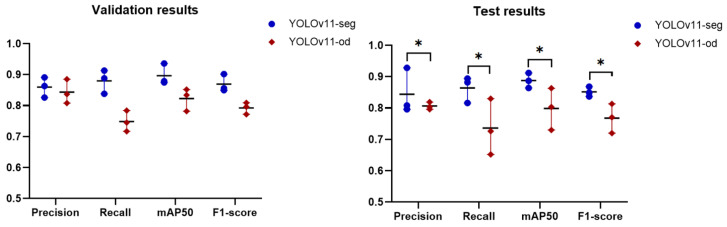
A scatter plot of performance comparison between object detection (YOLOv11-ob) and instance segmentation (YOLOv11-seg) tasks under IoU50 and confidence of 0.25. The asterisk (*) signifies a statistically significant enhancement attained by YOLOv11-seg, and the horizontal line denotes the mean of the three classes.

The results presented in Figure 9 demonstrated that the instance segmentation task consistently surpasses the object detection task in all classes and metrics. This indicates that instance segmentation is superior for tasks necessitating accurate localization and identification of several objects in images. The statistical analysis also showed a significant difference (*p* = 0.027) when using YOLOv11-seg.

These findings emphasize the benefits of instance segmentation in tasks where defining object boundaries is essential as in carious lesions, while also indicating contexts where object detection may remain effective, particularly regarding precision.

### 3.4. Results of Localizing Carious Lesion

**Figure 10:**
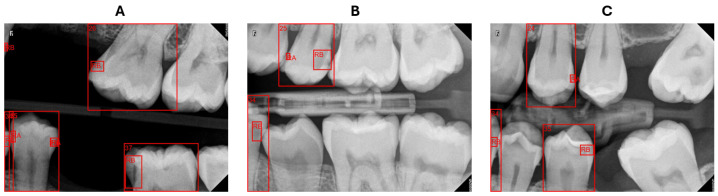
An example of incorrect identification of (**A**) the impacted tooth, (**B**,**C**) the impacted surface on the teeth that show up at the edges, where caries in tooth 34 was incorrectly designated as occurring on the mesial surface.

Out of 655 carious lesions of varying severities detected by the PCDM, the system successfully assigned tooth numbers to 652 lesions, while three lesions remained undetermined because they fell in an area that the tooth numbering model did not detect. This area is where the teeth partially appear at the BR edges, which is considered a challenging case in analyzing BRs, unlike a panoramic radiograph that offers a complete view of all teeth. For identifying the affected surfaces, 16 surfaces were incorrect due to the same reason as teeth at the edges, as illustrated in Figure 10. In this case, tooth 34 is partially visible and the caries is located on the distal surface. However, the dental caries was found to be on the mesial surface rather than the distal surface by comparing the x-midpoint of the caries to the x-midpoint of tooth 34. By disregarding this instance of teeth and their associated lesions, the accuracy in identifying impacted teeth and surfaces attains 100%.

**Figure 11:**
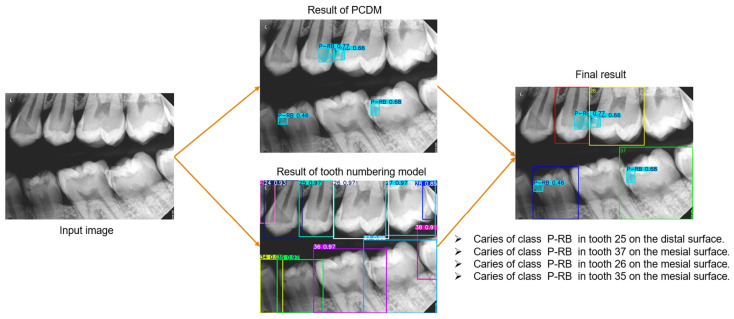
The phases of PCDS to obtain the final results.

The results of this module consider the final result of the PCDS and are presented as illustrated in Figure 11. In a comparison between seven general dentists and the proposed system (PCDS) on 10 randomly selected BRs from the test set, PCDS demonstrated superior performance, surpassing that of the dentists, as shown in Table 3.

## 4. Discussion

To the best of our knowledge, this is the first study utilizing YOLOv11 and offering a detailed description of the caries, encompassing its severity rating, the affected tooth, and the implicated surface. The proposed system attained exceptional performance, often equaling or surpassing that of dentists, rendering it a valuable tool for caries diagnosis.

The detection of caries encounters several challenges that hinder accurate diagnosis and treatment. The variety in appearance indicates that caries can manifest in many shapes, hues, and stages, complicating standardization. Moreover, the quality and sharpness of photographs may fluctuate, influencing the clarity of carious lesions. Overlapping structures in dental anatomy can conceal caries, resulting in false negatives or positives. Moreover, noise and abnormalities in dental pictures might hinder precise detection, and attaining real-time processing in clinical environments imposes further technical challenges. The research in the field has used DL methods to circumvent these problems [28]. Nevertheless, there is potential to improve the detection of caries.

This study aimed to create a novel diagnostic system for proximal caries from BRs. This system performs three critical tasks, see Figure 2, and the results were combined to provide the final diagnosis, with each task assigned to a distinct module to facilitate reuse in other applications. We addressed the tasks of detecting proximal caries of different depths and describing their location. The tooth numbering task was performed in our previous study [26], where the YOLOv8-od model was used and achieved 96.7% precision, 98% recall, and 97.2% mAP. This study presented and assessed various scenarios concerning caries detection, aiming to provide a comprehensive overview of the most effective methods for identifying caries. The YOLOv11-seg demonstrated superior performance compared to the YOLOv8-seg under the same setting of parameters. It is attributed to the improvements made in YOLOv11, which include the enhancement of the model’s capacity to detect smaller objects, which addresses a common challenge in object detection tasks. Additionally, the backbone and neck have been updated to improve feature extraction and aggregation, resulting in improved detection accuracy and efficiency. The model was able to optimize more effectively during training as a result of the updated loss functions, which resulted in a reduction in false positives and an increase in accuracy.

The findings of comparing the instance segmentation approach with object detection showed that YOLOv11-seg outperformed YOLOv11-od in all metrics, which can be attributed to the method’s mechanism. The instance segmentation method not only identifies objects but also defines their exact contours. It offers pixel-level segmentation, facilitating a more precise delineation of the caries’ boundaries. This comprehensive information aids in more efficiently differentiating between several classes, particularly when the objects are in proximity or overlap. Object detection finds objects and delineates them using bounding boxes, but it lacks specific shape information. This may result in mistakes when carious lesions are close or overlapping, leading to probable misclassification or undetected cases. Therefore, the choice between the two methods should be influenced by the particular objectives of the detection task, the resources at hand, and the requirement for diagnostic precision. In the recent studies [17,18] used YOLOv3 and YOLOv7, a caries was annotated by making a bounding box around the tooth it belonged to and its severity was assigned according to ICCMS^TM^. With an $IoU_{50}$, YOLO3 attained an mAP of 0.58 for detecting four classes (sound, RA, RB, RC), while YOLOv7 obtained 0.564 for seven classes (sound, RA1, RA2, RA3, RB4, RC5, RC6), and our model reached 0.888. Due of the imprecise marking of the caries, the models became sensitive to any dark areas such as pulp. This underscores the efficacy of the segmentation task in caries diagnosis.

This study addressed the influence of IoU and confidence thresholds on the task of caries detection. These thresholds play a crucial role in the YOLO network, significantly improving the evaluation and performance of object recognition. They enhance accuracy, regulate sensitivity, and guarantee that the model’s predictions are trustworthy and significant. Two values of IoU were studied, $IoU_{50}$ and $IOU_{75}$, and three levels of confidence thresholds, 0.001, 0.25, 0.5. For example, $IoU_{50}$ indicates at least 50% of the expected box must cross with the ground truth box to be regarded as a successful detection. The confidence threshold ranges from 0 to 1 and setting it to 0 incorporates all identified objects. For instance, with a criterion as minimal as 0.001, the model preserves all items exhibiting a confidence level exceeding 0.001, or 0.1%. This threshold may be useful in situations when catching as many objects as possible at the expense of accuracy is crucial. Example: surveillance systems that prioritize identifying every potential action over accuracy.

The findings underscore the trade-offs between precision and recall when adjusting IoU and confidence levels. In general, a lower IoU of 0.5, more than the higher IoU of 0.75, tended to boost both precision and recall, which, in turn, raised the value of mAP and F1-score as well. This indicated that the stricter threshold made it harder for the model to meet the criteria for TPs. In contrast, our previous model of tooth numbering achieved excellent performance with $IOU_{75}$, which emphasizes that the setting of these parameters depends on the object’s context. The annotation of caries is subjective, as annotators may encounter difficulty in precisely defining the boundaries of caries in contrast to the well-defined boundaries of teeth. As a result, the PCDM may accurately detect caries; however, the boundaries it identifies may not be precisely aligned with those in the reference dataset. Consequently, the IoU of 50% yielded superior outcomes compared to 75%.

The findings of variation of confidence thresholds revealed that lowering the confidence threshold increased the number of predictions, which can improve recall but could also lower precision because of more FPs. For instance, precision values are generally lower at a confidence level of 0.001 than at higher confidence levels, suggesting that many but not all predictions are made but not accurate. On the other hand, raising the confidence threshold resulted in reduced recall but greater precision. The model runs the danger of missing actual positive cases even as it becomes more discriminating about what it defines as a good prediction. For a confidence level of 0.5, for example, precision usually rises dramatically, but this comes at the cost of lower recall values. In fields like medical image analysis, high precision is crucial for achieving accurate diagnoses and treatments. An FP in medical imaging can lead to unnecessary interventions, whereas an FN may result in missed diagnoses, delayed treatments, or even harm to the patient. Therefore, this work proceeded with IoU50 and confidence of 0.25 to achieve a balance between accuracy and recall of 84.4% for precision and 86.4% for recall.

The PCDM demonstrated good recall for the initial stage (RA), indicating effective identification of many cases, which is crucial for early intervention. However, the precision of 0.796 suggests that it also includes a notable number of FPs. The model may have learned to recognize common patterns associated with initial caries, yet the complexity of distinguishing these subtle lesions from healthy enamel leads to some misclassifications. In addition, the overlapping teeth in the enamel region may produce shadows in the overlap area, which the model classified as RA in certain instances. In the moderate stage (RB), the high precision of 0.928 signifies that the model effectively detects the majority of cases, hence, reducing FPs. This is particularly important as timely detection can prevent further progression of caries. The recall of 0.816 indicates that certain mild cases are overlooked, perhaps due to the over-presence of class RB in the dataset, which may result in overfitting to particular features while disregarding other essential ones for identification. The extensive stage (RC) results demonstrate a balanced performance, with a high recall of 0.894, signifying the effective detection of class RC. A precision of 0.808 indicates a moderate degree of accuracy, implying that the model effectively identifies advanced caries but may erroneously identify certain non-caries cases, such as fractured and damaged teeth, as caries. Overall, these findings demonstrate the model’s promise as a dental diagnostic tool that can improve the accuracy of proximal caries detection and treatment planning.

The BRs are intended to concentrate on particular areas of the dental arch; however, they may depict teeth that are only partially visible or not entirely captured in the image. Our system struggled with these cases of teeth either in numbering teeth or determining the affected surface. In certain instances, as presented in Figure 10A, the system identified the caries but failed to recognize the corresponding tooth, rendering it incapable of describing this caries’ location. When determining the affected surface, the x-midpoint of a tooth does not reflect the actual half of the tooth, as illustrated in Figure 10B,C tooth 34, resulting in an incorrect surface assignment. However, teeth partially visible at the edges of radiography pictures are not a major issue for the dentist, who prioritizes obtaining comprehensive images of the pertinent teeth for precise evaluation and diagnosis.

Compared to previous studies utilizing deep learning algorithms for caries detection in dental radiographs, our results exhibit competitive efficacy. For instance, the study by Ayhan et al. [20] showcases a caries detection F1-score of 0.822, alongside precision and recall values of 0.866 and 0.833, respectively. While our precision is slightly lower than theirs, our recall is higher, indicating that our model is more effective at identifying TP cases of caries. This enhanced recall suggests that our approach may be more adept at minimizing FNs, which is critical in clinical settings where timely intervention is essential.

The study by Panyarak et al. [17] evaluated the YOLOv3 model for caries detection based on the ICCMS^TM^. The precision and recall values for the RA category in the current study are 0.796 and 0.882, respectively, which significantly exceed those reported by Panyarak et al., who found values of 0.75 for precision and 0.67 for recall. In the RB category, the metrics of the current study (0.928 precision and 0.816 recall) demonstrate enhanced performance relative to the YOLOv3 model, which achieved 0.62 precision and 0.53 recall. The RC category demonstrated superior metrics in the current study, with precision at 0.808 and recall at 0.894, compared to the previous study.

The findings of this study indicate improved efficacy in caries detection across all severity levels relative to the earlier research. Advancements in model architecture and instance segmentation techniques, exemplified by YOLOv11, may significantly enhance diagnostic accuracy in dental radiology.

Although the system attained exceptional performance, it is limited to diagnosing proximal caries and BRs from one source. As a consequential work, we aim to incorporate more caries types, such as occlusal caries, and gather data from diverse sources and populations to enhance the generalizability of the system.

## 5. Conclusions

In this research, we proposed a novel proximal caries diagnostic system that detects and diagnoses caries from BRs. The system demonstrated high sensitivity and accuracy, effectively identifying and diagnosing carious lesions in comparison to dentist evaluations. Caries detection was performed using a YOLOv11-based instance segmentation model, which demonstrated a robust capability in identifying caries of varying severity, particularly at an IoU of 50% and a confidence threshold of 0.25. Different scenarios were presented and evaluated. The findings indicated that stricter thresholds generally improve precision but can lead to reduced recall, making it essential to find a balanced setting based on the clinical context. The segmentation method outperforms the object detection method in the context of detecting caries. With the promising results the system achieved, it can be incorporated into practical use to help dentists and enhance proximal caries diagnosis. To improve the system’s usability in any location, data from various sources will be incorporated into future work. Additionally, additional improvements will be implemented to enhance the system’s power.

## Figures and Tables

**Table 1:** Quantitative statistics of the data for each category and dataset.

	Original Data					After Augmentation				
Dataset	Images	RA	RB	RC	Total	Images	RA	RB	RC	Total
Training	1084	1371	3275	542	5188	3198	4061	9703	1596	15,360
Validation	135	234	331	66	631	403	679	989	196	1864
Testing	135	221	424	47	692	135	221	424	47	692
Total	1354	1826	4030	655	6511	3736	4961	11,116	1839	17,916

**Table 2:** The Performance of PCDM using $IoU_{50}$ and conf = 0.25.

	Precision	Recall	mAP50	F1-Score
RA	0.796	0.882	0.864	0.837
RB	0.928	0.816	0.887	0.868
RC	0.808	0.894	0.912	0.849
Overall performance	0.844	0.864	0.888	0.851

**Table 3:** Performance evaluation of the PCDS against seven dentist assessments in caries diagnosis.

A Total Instance of Caries = 62	PCDS	Dentist1	Dentist 2	Dentist 3	Dentist 4	Dentist 5	Dentist 6	Dentist 7
Detected instances	61	22	27	17	22	19	13	27
Correct instances	58	12	18	2	14	14	8	14
Determine severity correctly	59	13	18	10	16	15	8	17
Determine tooth correctly	61	22	26	2	18	19	13	25
Determine surface correctly	60	21	26	15	22	18	13	26

Correct instances mean the complete diagnosis is correct, including caries severity and the affected tooth and surface.

## Data Availability

The datasets presented in this article are not readily available due to ethical limitations. Requests to access the datasets should be directed to the University Dental Hospital of King Abdulaziz University.
